# Prevalence of Psychological Impacts on Healthcare Providers during COVID-19 Pandemic in Asia

**DOI:** 10.3390/ijerph18179157

**Published:** 2021-08-30

**Authors:** Mohd Noor Norhayati, Ruhana Che Yusof, Mohd Yacob Azman

**Affiliations:** 1 Department of Family Medicine, School of Medical Sciences, Universiti Sains Malaysia, Kubang Kerian 16150, Malaysia; hayatikk@usm.my; 2 Federal Government Administrative Centre, Medical Practice Division, Ministry of Health, Level 7, Block E1, Parcel E, Putrajaya 62590, Malaysia; drmohdazman@moh.gov.my

**Keywords:** COVID-19, psychological impacts, anxiety, depression, stress, insomnia, PTSD, prevalence, systematic review

## Abstract

COVID-19 has impacted people psychologically globally, including healthcare providers. Anxiety, depression, and stress are the most common impacts that have affected these people. Thus, this study was aimed to ascertain the estimated prevalence of psychological impacts among healthcare providers in the Asian region. A systematic search was performed in the MEDLINE, CINAHL, and Scopus databases for original research articles published between 2020 and April 2021. Only studies published in English were included. The quality of data was assessed using the Joanna Briggs Institute Meta-Analysis, and the analysis was performed using generic inverse variance with a random-effects model by Review Manager software. A total of 80 studies across 18 countries in Asia region were pooled to assess the data prevalence on anxiety (34.81% (95% CI: 30.80%, 38.83%)), depression (34.61% (95% CI: 30.87%, 38.36%)), stress (31.72% (95% CI: 21.25%, 42.18%)), insomnia (37.89% (95% CI: 25.43%, 50.35%)), and post-traumatic stress disorder (15.29% (95% CI: 11.43%, 19.15%)). Subgroup analyses were conducted across regions, type of healthcare providers, sex, and occupation. This review has identified a high prevalence of anxiety, depression, stress, and insomnia but a low prevalence of post-traumatic stress disorder among healthcare providers in Asia regions. Effective intervention support programs are urgently needed to improve psychological health of healthcare providers and maintaining the health system.

## 1. Introduction

Psychological impacts refer to the effect caused by environmental and/or biological factors on an individual’s social and/or psychological aspects [1]. COVID-19, caused by severe acute respiratory syndrome coronavirus 2 (SARS-CoV-2), has drastically spread worldwide [2]. The sudden pandemic of COVID-19 has affected healthcare providers physically and psychologically by dramatically increasing the number of patients infected with the disease, which impacted changes in the working environment [3].

Healthcare providers were reported to have more severe psychological impacts than the general population [4,5]. However, a review study [6] reported that the pooled prevalence of anxiety and depression in the general population was higher than healthcare providers. Healthcare providers were psychologically burdened with the responsibility to face challenges in treating COVID-19 patients, reducing the infection trend, developing and formulating strategies and plans in combating this pandemic [7]. Healthcare providers, especially frontline employees who were directly exposed to COVID-19 patients, had more mental problems than those who were not directly involved with COVID-19 patients [8,9]. It may be influenced by physical and mental stress due to the rapidly increasing number of infected COVID-19 patients, increased workload, burnout, increased number of infected colleagues, and lack of contact with their families [8].

The common psychological impacts that affected healthcare providers during a pandemic or an outbreak were anxiety and depression [10,11,12], trauma, and post-traumatic stress disorder (PTSD) [13,14] and insomnia [15,16]. The level of psychological impacts was reported higher during COVID-19 than during Middle East respiratory syndrome coronavirus (MERS-CoV) or seasonal influenza [17]. Undeniable, the outbreak of COVID-19, MERS-CoV, and even Severe Acute Respiratory Syndrome (SARS) substantially impacts healthcare providers’ psychological health [18].

Many studies have been reported the prevalence of these psychological impacts among healthcare providers. A review study of several regions in the world had determined the pooled estimated prevalence of moderate depression in East Asia (19.1% (95% CI: 15.2%, 23.4%)), Middle East (34.6% (95% CI: 25.1%, 44.9%)) Europe (22.0% (95% CI: 18.9%, 25.3%)), South East (28.8% (95% CI: 18.1%, 40.8%)) and North America (18.7% (95% CI: 17.8%, 19.7%)). The pooled prevalence of depression and anxiety was 40% (95% CI: 19%, 62%) and 38% (95% CI: 12%, 63%), respectively, before the peak of COVID-19 pandemic but decreased to 22% (95% CI: 13%, 31%) and 22% (95% CI: 13%, 31%), respectively, after the peak [5]. Meanwhile, the pooled prevalence for PTSD among healthcare providers was 21.5% (95% CI: 10.5%, 34.9%) [19]. For insomnia, the overall prevalence was 27.8% (95% CI: 21.4%, 35.3%) and it was higher in nurses compared to doctors (42.4% vs. 39.1%) [20].

Determining the pooled prevalence of psychological impacts among healthcare providers gives a better figure than discrete primary studies. It serves as a basis for an appropriate preventive strategy to be established. It applies to primary prevention at the institutional, provider, and client levels by screening or prevention that could potentially prevent a condition from mental health illness. This systematic review was aimed to ascertain the prevalence of psychological impacts among healthcare providers in the Asian region.

## 2. Materials and Methods

### 2.1. Study Design and Search Strategy

A systematic review and meta-analysis were conducted to assess the prevalence of psychological impacts among healthcare providers in the Asian region (PROSPERO registration number: CRD42021247747). The interesting psychological impacts were anxiety, depression, stress, insomnia, and PTSD. The guidelines of preferred reporting items for systematic reviews and meta-analyses (PRISMA) [21] were followed.

A systematic search was performed in the MEDLINE (PubMed), CINAHL (EBSCOhost) and Scopus databases for articles between 1 January 2020 and 15 April 2021. The search was done using the Medical Subject Headings (MeSH) search terms: “prevalence”. Generic free-text search terms, synonymous with “psychological impact” such as “psychological distress”, “psychological disturbance”, “anxiety”, “depression”, “vicarious traumatization”, “secondary traumatic stress” AND “COVID-19”, “coronavirus”, “2019-ncov”, “sars-cov-2” AND “healthcare providers”, “healthcare professional”, “healthcare workers” AND “Asia” was used.

The search terms were flexible and tailored to various electronic databases. All studies published from 2020 were retrieved to assess their eligibility for inclusion in this study. The search will be restricted to full-text and English language articles. To find additional potentially eligible studies, reference lists of included citations were cross-checked.

### 2.2. Eligibility Criteria

The inclusion criteria of selected studies involved the reported prevalence of psychological impacts (anxiety, depression, stress, insomnia, and PTSD) among healthcare providers in the Asian country. Studies with cross-sectional, case-control, and cohort designs published in the English language (abstract and full text) were included. Case report, conference papers, proceeding, editorial reviews, letters of communication, commentaries, systematic reviews, and qualitative studies were excluded.

### 2.3. Study Selection and Screening

**Figure 1:**
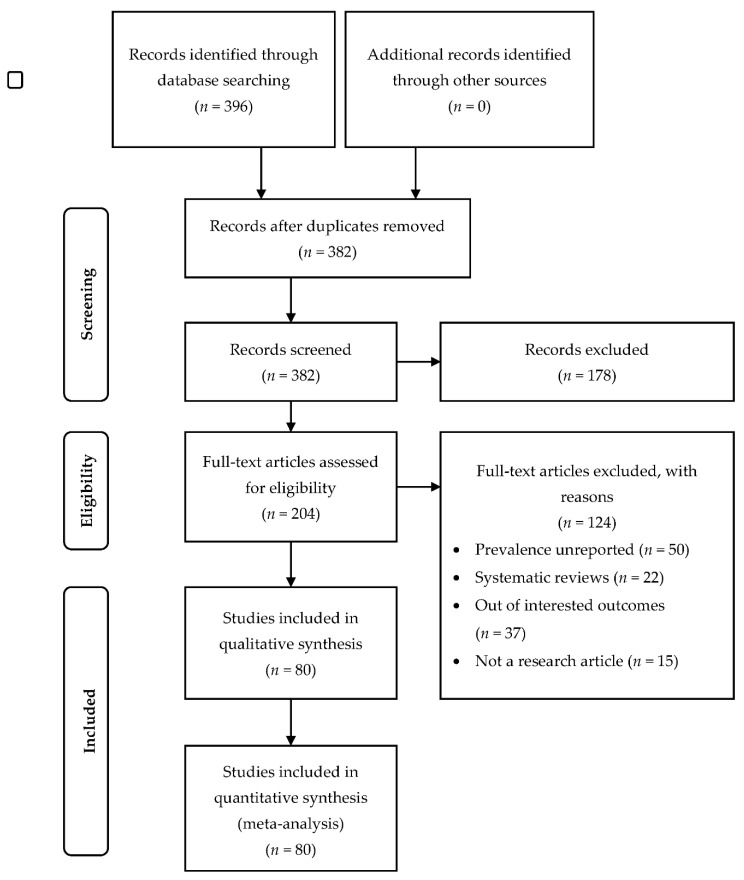
PRISMA flow chart of the review.

All records identified by our search strategy were exported to EndNote software. Duplicate articles were removed. A reviewer screened the titles and abstracts of the identified articles. The full texts of eligible studies were obtained and read thoroughly to assess for their suitability. These processes including evaluation risk of bias and data extraction were verified by a second reviewer. In the event of a conflict between the two reviewers, a consensus discussion was held, and a third reviewer was consulted. The search method was presented in the PRISMA flow chart showing the studies that are included and excluded with reasons for exclusion (Figure 1).

### 2.4. Quality Assessment and Bias

A critical appraisal was done to assess the data quality by using the Joanna Briggs Institute Meta-Analysis for cross-sectional, case-control, and cohort studies [22]. Two reviewers performed bias assessments independently. The risk of bias was considered low when more than 70% of the answers were “yes,” moderate when 50–69% of the answers were “yes,” and high when up to 49% of the answers were “yes.” “Yes” was score as 1 and “No” was score as 0.

The risk of bias was assessed by nine criteria [23]: (1) Was the sample frame appropriate to address the target population? (2) Were study participants sampled in an appropriate way? (3) Was the sample size adequate? (4) Were the study subjects and the setting described in detail? (5) Was a sample size justification, power description, or variance and effect estimates provided? (6) Were valid methods used for the identification of the condition? (7) Was the condition measured in a standard, reliable way for all participants? (8) Was there appropriate statistical analysis? (9) Was the response rate adequate, and if not, was the low response rate managed appropriately?

### 2.5. Data Extraction

The data were extracted into Microsoft Excel, including first author, year of publication, study location, study design, setting, study population, sample size, prevalence, and data for calculation of effect estimates.

### 2.6. Data Synthesis and Analysis

The outcomes were reported as prevalence or proportion of total sample over total population. For this study and ease the interpretation, the psychological impacts were defined as a combination of the symptoms such as mild, and/or mild to moderate, and/or moderate, and/or moderate to severe, and/or severe as defined by the authors since the assessment tools were varies for each study and were categorized differently.

The analysis was performed with Review Manager software version 5.4 (Nordic Cochrane Centre). A generic inverse variance with a random-effects model was used to pool the data. The I^2^ statistic was used to assess heterogeneity and used the guide as outlined: 0% to 40% might not be important, 30% to 60% may represent moderate heterogeneity, 50% to 90% may represent substantial heterogeneity, and 75% to 100% would be considerable heterogeneity [24]. Funnel plots were used to assess publication bias visually if indicated.

A subgroup analysis was performed based on the Asia region (Central Asia, Eastern Asia, South-Eastern Asia, Southern Asia, and Western Asia), type of healthcare (frontline and non-frontline), sex (female and male), and occupation (doctors and nurses). Subgroup analysis was completed to explore and compare the pooled prevalence of different subgroups [25]. The prevalence of the psychological impacts might be varying across the subgroup.

## 3. Results

### 3.1. Searching Results and Study Characteristics

The initial search had identified 396 potentially relevant studies. A total of 14 duplicated studies was removed, and 382 studies were screened. After the screening of titles and abstracts, 178 studies were excluded. A total of 204 full-text articles were screened. In total, 124 studies were excluded due to unreported prevalence, systematic reviews, not reporting the outcomes, and not in the form of research articles. As a result, a total of 80 studies that met the inclusion and exclusion criteria was included in this review. The articles were published in 2020 (*n* = 56) and in early 2021 (*n* = 24) (Figure 1).

Data quality assessment had shown that 18 studies were low risk of bias, 51 studies were moderate bias, and 11 studies were high risk of bias. All these studies were included in the analysis (Supplementary Table S1).

The studies were from Eastern Asia (*n* = 40) [8,9,10,26,27,28,29,30,31,32,33,34,35,36,37,38,39,40,41,42,43,44,45,46,47,48,49,50,51,52,53,54,55,56,57,58,59,60,61,62], South-eastern Asia (*n* = 5) [63,64,65,66,67], Southern Asia (*n* = 18) [68,69,70,71,72,73,74,75,76,77,78,79,80,81,82,83,84,85], Western Asia (*n* = 15) [4,17,86,87,88,89,90,91,92,93,94,95,96,97,98] and combination of South-eastern and Southern Asia (*n* = 2) [99,100] (Supplementary Table S2). Studies from Eastern Asia came from China, Japan, and Korea. Meanwhile, for South-Eastern Asia, the studies were from Indonesia, Malaysia, Singapore, and Vietnam. India, Bangladesh, Iran, Nepal, Pakistan, and Sri Lanka were represented in Southern Asia and Western Asia included studies from Jordan, Kuwait, Oman, Saudi Arabia, Turkey, and Yemen. No study from Central Asia was found. A total of 149,925 healthcare providers across 18 countries in Asia participated in these studies. Subgroup studies involved type of healthcare (*n* = 13) [8,26,28,29,39,46,52,61,66,77,82,83,95], sex (*n* = 27) [10,26,27,29,30,32,39,42,52,53,54,60,66,68,69,71,74,76,77,83,84,87,88,94,95,96,98] and occupation (*n* = 21) [27,29,39,40,42,45,52,53,54,66,73,74,75,82,83,84,85,87,94,95,98].

Most of the studies were cross-sectional in design (*n* = 78). One was a matched case-control [25] and one was a longitudinal [28] study. The majority of the studies applied convenience sampling as a sampling method. Some of the studies did not mention the sampling methods.

### 3.2. Prevalence of Anxiety

The overall pooled prevalence of anxiety for the 68 studies was 34.81% (95% CI: 30.80%, 38.83%) and varies from 5.12% [57] to 100% [67,79]. Western Asia showed the highest prevalence of anxiety (46.57% (95% CI: 33.34%, 59.80%)) and the lowest prevalence of anxiety was South-Eastern Asia (24.91% (95% CI: 10.51%, 39.05%)). Non-frontline healthcare providers (24.35% (95% CI: 18.71%, 29.99%)), female (36.14% (95% CI: 22.50%, 49.78%)) and nurses (36.06% (95% CI: 23.75%, 48.38%)) had higher prevalence in the subgroup analysis. All data had considerable heterogeneity (I^2^ > 75%) (Table 1).

### 3.3. Prevalence of Depression

The pooled prevalence of depression in this review was 34.61% (95% CI: 30.87%, 38.36%) involved 60 studies. Western Asia (43.78% (95% CI: 28.36%, 59.19%)) showed the highest prevalence of depression and the lowest was South-Eastern Asia (22.96% (95% CI: 13.23%, 32.68%)). The depression was highest in frontline healthcare providers, females, and nurses. All data had considerable heterogeneity (I^2^ > 75%) (Table 2).

### 3.4. Prevalence of Stress

The overall pooled prevalence from 20 studies was 31.72% (95% CI: 21.25%, 42.18%). The highest prevalence of stress was in Western Asia (48.97% (95% CI: 30.67%, 67.28%)) and the lowest was in Eastern Asia (19.42% (95% CI: −4.88%, 43.72%)). The prevalence was also higher in frontline healthcare providers, females, and nurses in subgroup analysis. All the data had considerable heterogeneity (I^2^ > 75%) (Table 3).

### 3.5. Prevalence of Insomnia

The pooled prevalence of insomnia was 37.89% (95% CI: 25.43%, 50.35%) from 12 studies. The highest prevalence of stress was identified in Eastern Asia (41.23% (95% CI: 19.75%, 62.70%)) and the lowest was in Southern Asia (31.30% (95% CI: 17.82%, 44.78%)). The prevalence was also higher in frontline healthcare providers, females, and nurses in subgroup analysis. All the data had considerable heterogeneity (I^2^ > 75%) except for frontline that had substantial heterogeneity (I^2^ > 64%). Heterogeneity for occupation was not available since the data only involved one study (Table 4).

### 3.6. Prevalence of Post-Traumatic Stress Disorder (PTSD)

The pooled prevalence of PTSD was 15.29% (95% CI: 11.43%, 19.15%). Eastern Asia (17.61% (95% CI: 12.59%, 22.62%)) was the highest in prevalence of PTSD and Southern Asia (4.58% (95% CI: −4.51%, 9.66%)) was the lowest in the prevalence. The prevalence were also higher in frontline healthcare providers, females, and nurses in subgroup analysis. All the data had considerable heterogeneity (I^2^ > 75%) except for females, where heterogeneity might not be important (I^2^ = 0%) (Table 5).

### 3.7. Prevalence of Fear

There were two studies that reported fear. The prevalence of fear was 100.0% [70] and 69.2% [41]. The prevalence of 100.0% was not estimable; hence, the prevalence of fear in this review was 69.2% (95% CI: (67.32, 71.09)).

## 4. Discussion

A high prevalence of psychological impacts was identified among healthcare providers in Asia. These psychological impacts were associated with work-related stress, a potential cause of concern in healthcare providers [101]. More than 30% of healthcare providers were affected by anxiety, depression, stress, and insomnia during the COVID-19 pandemic. Meanwhile, 15% of healthcare providers experienced PTSD. This prevalence was similar to a review of 50 studies [102] but higher compared to previous reviews [6,19,20] and lower than another review of 40 studies among nurses [103]. These current prevalence of psychological impacts were lower if compared to the pooled prevalence of these psychological impacts outside the Asia region [20]. Unfortunately, no data for insomnia and PTSD were available in the previous review to compare with. The another review [19] reported that the pooled prevalence of anxiety and depression in Europe and North America was lower than the current review, but that review used a moderate level of anxiety and depression as outcomes.

In this review, Western Asia showed the highest prevalence of anxiety, depression, and stress, but no data are available on PTSD. As reported in a study from Saudi Arabia, 68.5% of healthcare providers presented with moderate and severe anxiety [87], 55.2% had depressive disorder [86], and 55.9% had stress [92] during the COVID-19 pandemic. Meanwhile, Eastern Asia showed the highest prevalence of insomnia and PTSD but the lowest prevalence of stress. Previous studies showed that 34–36.1% of healthcare providers had insomnia in China [104,105], and 42.2% in Japan [106]. On the other hand, PTSD in China range between 9.8% to 31.6% [37,52] and 20.3% in Korea [107]. South-Eastern Asia showed the lowest prevalence of anxiety (6.6% to 49.6%) and depression (6.3% to 41%) but was unknown for insomnia since no data were available to review. A meta-analysis showed that the pooled prevalence of anxiety and depression in South-Eastern Asia was 22% and 16%, respectively [108]. Southern Asia had the lowest prevalence of insomnia (18.6% to 41.3%) and PTSD (2.1% to 7.3%) which was similar in a previous study [100].

Many studies had compared the psychological impacts between frontline and non-frontline healthcare providers. A systematic review and meta-analysis [109] suggested that frontline healthcare providers suffer more psychological impacts such as sleep disturbance, anxiety, PTSD, and burnout than non-frontline healthcare providers. The review also revealed that non-frontline had a more severe psychological problem than frontline, but some of the studies did not significantly differ between the groups. In this review, frontline healthcare providers showed the higher prevalence of depression, stress, insomnia, and PTSD compared to non-frontline healthcare providers. For anxiety, the prevalence between both groups was similar with 0.33% higher in non-frontline healthcare providers. This finding was similar to studies in Malaysia [110] and Singapore [111] but contradicted with a study from Taiwan [112]. A review from a Western country had shown that the frontline healthcare providers who were directly in contact with COVID-19 patients had the greatest impacts psychologically that were worsened by long working hours and contacted with suffering and death [113].

This review proved that female healthcare providers showed a higher prevalence of all psychological impacts than males. A few reviews also showed similar findings [108,113,114]. The recommendation to include a gender perspective in the planning and designing support measures and intervention was widely documented. It was suggested to reduce psychological problems due to the high feminization of the health sector and increased risk and exposure to psychological health problems among female healthcare providers [113].

Nurses are well-known to be affected psychologically during the COVID-19 pandemic. The prevalence also proved it in this review. The prevalence of psychological impacts (anxiety, depression, stress, insomnia, and PTSD) was higher among nurses than doctors. A previous review suggested that at least one-third of nurses have experienced psychological impacts [103]. A longitudinal study [28] also showed that nurses significantly suffered more psychological impacts during an outbreak than those in stable periods. A survey in the Philippines has determined that the psychological impact was lower by personal resilience and social and organizational support [115].

The strengths of this review included the estimates of pooled prevalence of anxiety, depression, stress, insomnia, and PTSD. The large sample size involving 149,925 healthcare providers in 80 studies. The included studies were from 18 countries across every region in Asia. Unfortunately, there was no study found for Central Asia. Subgroup analysis was made from the data of the studies and was categorized based on Asia regions, type of healthcare, sex, and occupation.

This review is not without limitations. The search was limited to the English language only. Besides that, various psychological impacts tools were used involving different cut off points, scales, and classifications. To synchronize the classification, the authors had to include all classifications such as mild, mild to moderate, moderate, moderate to severe, and severe. They exclude the no symptoms, or normal classification, then set it as a psychological impact. Misclassification might happen in this process. To have a more comprehensive range of the data, the authors decided to include all the available studies even the quality of the data were low, moderate, or high based on the risk of bias assessment.

## 5. Conclusions

High prevalence of anxiety, depression, stress, and insomnia but low prevalence of PTSD was identified among healthcare providers in Asia included the Eastern region, South-Eastern region, Southern region, and Western region. It might become worse with the emergence of new variants of the virulent COVID-19 virus. Effective intervention support programs are urgently needed to improve healthcare providers’ psychological health and maintain the health system in combating the COVID-19 pandemic. The intervention should be focused more specifically on affected groups such as frontline healthcare providers, females, and nurses.

## Figures and Tables

**Table 1:** Pooled prevalence of anxiety and its subgroup analysis.

Outcome		No. of Studies	No. of Participants	Prevalence [95% CI]	I^2^ (%)	*p*-Value
Overall anxiety		68	124,925	34.81 [30.80, 38.83]	100	*p* < 0.001
**Subgroup**						
Regions	Eastern Asia	35	90,039	28.99 [24.91, 33.07]	100	*p* < 0.001
	South-Eastern Asia	7	13,140	24.78 [10.51, 39.05]	100	*p* < 0.001
	Southern Asia	14	5449	38.42 [22.89, 53.94]	100	*p* < 0.001
	Western Asia	14	16,297	46.57 [33.34, 59.80]	100	*p* < 0.001
Type of healthcare provider	Frontline	13	7816	24.02 [15.89, 32.16]	99	*p* < 0.001
	Non-frontline	13	13,619	24.35 [18.71, 29.99]	99	*p* < 0.001
Sex	Female	21	19,800	36.14 [22.50, 49.78]	100	*p* < 0.001
	Male	21	7788	32.76 [18.16, 47.36]	100	*p* < 0.001
Occupation	Doctors	18	7279	30.10 [20.56, 39.64]	99	*p* < 0.001
	Nurses	18	11,574	36.06 [23.75, 48.38]	100	*p* < 0.001

**Table 2:** Pooled prevalence of depression and subgroup analysis.

Outcome		No. of Studies	No. of Participants	Prevalence [95% CI]	I^2^ (%)	*p*-Value
Overall depression		60	132,308	34.61 [30.87, 38.36]	100	*p* < 0.001
**Subgroup**						
Regions	Eastern Asia	33	103,868	31.47 [26.88, 36.05]	99	*p* < 0.001
	South-Eastern Asia	7	13,140	22.96 [13.23, 32.68]	98	*p* < 0.001
	Southern Asia	13	6563	38.51 [22.77, 54.26]	98	*p* < 0.001
	Western Asia	9	8737	43.78 [28.36, 59.19]	99	*p* < 0.001
Type of healthcare provider	Frontline	12	7656	32.69 [19.55, 45.84]	100	*p* < 0.001
	Non-frontline	12	13,458	28.76 [20.43, 37.09]	99	*p* < 0.001
Sex	Female	22	18,306	32.32 [24.36, 40.28]	100	*p* < 0.001
	Male	22	7648	27.36 [20.31, 34.41]	99	*p* < 0.001
Occupation	Doctors	19	6845	28.34 [18.93, 37.75]	99	*p* < 0.001
	Nurses	19	10,159	36.64 [27.10, 46.18]	99	*p* < 0.001

**Table 3:** Pooled prevalence of stress and subgroup analysis.

Outcome		No. of Studies	No. of Participants	Prevalence [95% CI]	I^2^ (%)	*p*-Value
Overall stress		20	12,673	31.72 [21.25, 42.18]	100	*p* < 0.001
**Subgroup**						
Regions	Eastern Asia	4	3870	19.42 [−4.88, 43.72]	98	*p* < 0.001
	South-Eastern Asia	3	1635	28.23 [10.50, 45.96]	95	*p* < 0.001
	Southern Asia	7	2885	31.41 [13.46, 49.35]	95	*p* < 0.001
	Western Asia	6	5559	48.97 [30.67, 67.28]	99	*p* < 0.001
Type of healthcare provider	Frontline	2	647	41.51 [−29.76, 112.79]	100	*p* < 0.001
	Non-frontline	2	407	38.39 [−31.53, 108.31]	100	*p* < 0.001
Sex	Female	5	1105	59.96 [31.01, 88.92]	99	*p* < 0.001
	Male	5	852	42.46 [17.56, 67.37]	99	*p* < 0.001
Occupation	Doctors	2	688	61.08 [32.79, 89.37]	97	*p* < 0.001
	Nurses	2	315	79.19 [72.88, 85.50]	30	0.230

**Table 4:** Pooled prevalence of insomnia and subgroup analysis.

Outcome		No of Studies	No of Participants	Prevalence [95% CI]	I^2^ (%)	*p*-Value
Overall insomnia		12	14,877	37.89 [25.43, 50.35]	100	*p* < 0.001
**Subgroup**						
Regions	Eastern Asia	6	10,909	41.23 [19.75, 62.70]	100	*p* < 0.001
	South-Eastern Asia	0		NA		
	Southern Asia	3	1783	31.30 [17.82, 44.78]	89	0.0001
	Western Asia	3	2185	37.80 [13.20, 62.40]	98	*p* < 0.001
Type of healthcare provider	Frontline	3	2090	49.27 [45.42, 53.12]	64	0.060
	Non-frontline	3	1904	35.10 [25.39, 44.81]	94	*p* < 0.001
Sex	Female	3	1402	51.61 [26.75, 76.47]	99	*p* < 0.001
	Male	3	735	44.16 [8.05, 80.26]	99	*p* < 0.001
Occupation	Doctors	1	161	29.19 [22.17, 36.22]	NA	
	Nurses	1	167	41.92 [34.43, 49.40]	NA	

**Table 5:** Pooled prevalence of PTSD and subgroup analysis.

Outcome		No of Studies	No of Participants	Prevalence [95% CI]	I^2^ (%)	*p*-Value
	Overall PTSD	9	23,012	15.29 [11.43, 19.15]	99	*p* < 0.001
**Subgroup**						
Regions	Eastern Asia	7	20,960	17.61 [12.59, 22.62]	99	*p* < 0.001
	South-Eastern Asia	2	1242	9.73 [5.38, 14.08]	86	0.008
	Southern Asia	2	810	4.58 [−0.51, 9.66]	92	0.0004
	Western Asia	0		NA		
Type of healthcare provider	Frontline	3	1249	22.86 [11.39, 34.32]	95	*p* < 0.001
	Non-frontline	3	1679	15.81 [4.61, 27.00]	96	*p* < 0.001
Sex	Female	2	2300	10.81 [9.54, 12.07]	0	0.440
	Male	2	688	8.12 [4.41, 11.82]	70	0.070
Occupation	Doctors	2	688	9.65 [3.22, 16.07]	76	0.040
	Nurses	2	1591	13.85 [7.46, 20.25]	85	0.009

## Data Availability

Data are contained within the article.

## Supplementary Materials

The following are available online at www.mdpi.com/article/10.3390/ijerph18179157/s1, Table S1: JBI Quality Assessment Tool for prevalence studies, Table S2: Studies characteristics.

## References

[1] de Oliveira A.M., Buchain P.C., Vizzotto A.D.B., Elkis H., Cordeiro Q., Gellman M.D., Turner J.R. (2013). Psychosocial Impact. Encyclopedia of Behavioral Medicine.

[2] Salzberger B., Buder F., Lampl B., Ehrenstein B., Hitzenbichler F., Holzmann T., Schmidt B., Hanses F. (2021). Epidemiology of SARS-CoV-2. Infection.

[3] Coto J., Restrepo A., Cejas I., Prentiss S. (2020). The impact of COVID-19 on allied health professions. PLoS ONE.

[4] Naser A.Y., Dahmash E.Z., Al-Rousan R., Alwafi H., Alrawashdeh H.M., Ghoul I., Abidine A., Bokhary M.A., Al-Hadithi H.T., Ali D. (2020). Mental health status of the general population, healthcare professionals, and university students during 2019 coronavirus disease outbreak in Jordan: A cross-sectional study. Brain Behav..

[5] Deng Y., Chen Y., Zhang B. (2021). Different prevalence trend of depression and anxiety among healthcare workers and general public before and after the peak of COVID-19 occurred in China: A meta-analysis. Asian J. Psychiatry.

[6] Luo M., Guo L., Yu M., Jiang W., Wang H. (2020). The psychological and mental impact of coronavirus disease 2019 (COVID-19) on medical staff and general public—A systematic review and meta-analysis. Psychiatry Res..

[7] Shreffler J., Petrey J., Huecker M. (2020). The Impact of COVID-19 on Healthcare Worker Wellness: A Scoping Review. West. J. Emerg. Med..

[8] Cai Q., Feng H., Huang J., Wang M., Wang Q., Lu X., Xie Y., Wang X., Liu Z., Hou B. (2020). The mental health of frontline and non-frontline medical workers during the coronavirus disease 2019 (COVID-19) outbreak in China: A case-control study. J. Affect. Disord..

[9] Liu C.Y., Yang Y.Z., Zhang X.M., Xu X., Dou Q.L., Zhang W.W., Cheng A.S.K. (2020). The prevalence and influencing factors in anxiety in medical workers fighting COVID-19 in China: A cross-sectional survey. Epidemil. Infect..

[10] Yang S., Kwak S.G., Ko E.J., Chang M.C. (2020). The Mental Health Burden of the COVID-19 Pandemic on Physical Therapists. Int. J. Environ. Res. Public Health.

[11] Zhang W.R., Wang K., Yin L., Zhao W.F., Xue Q., Peng M., Min B.Q., Tian Q., Leng H.X., Du J.L. (2020). Mental Health and Psychosocial Problems of Medical Health Workers during the COVID-19 Epidemic in China. Psychother. Psychosom..

[12] Cabarkapa S., Nadjidai S.E., Murgier J., Ng C.H. (2020). The psychological impact of COVID-19 and other viral epidemics on frontline healthcare workers and ways to address it: A rapid systematic review. Brain Behav. Immun. Health.

[13] Zvolensky M.J., Garey L., Rogers A.H., Schmidt N.B., Vujanovic A.A., Storch E.A., Buckner J.D., Paulus D.J., Alfano C., Smits J.A.J. (2020). Psychological, addictive, and health behavior implications of the COVID-19 pandemic. Behav. Res. Ther..

[14] Sarapultseva M., Zolotareva A., Kritsky I., Nasretdinova N., Sarapultsev A. (2021). Psychological Distress and Post-Traumatic Symptomatology Among Dental Healthcare Workers in Russia: Results of a Pilot Study. Int. J. Environ. Res. Public Health.

[15] Babicki M., Szewczykowska I., Mastalerz-Migas A., Galeazzi G.M. (2021). Mental Health in the Era of the Second Wave of SARS-CoV-2: A Cross-Sectional Study Based on an Online Survey among Online Respondents in Poland. Int. J. Environ. Res. Public Health.

[16] Wu K., Wei X. (2020). Analysis of Psychological and Sleep Status and Exercise Rehabilitation of Front-Line Clinical Staff in the Fight Against COVID-19 in China. Med. Sci. Monit. Basic Res..

[17] Temsah M.H., Al-Sohime F., Alamro N., Al-Eyadhy A., Al-Hasan K., Jamal A., Al-Maglouth I., Aljamaan F., Al Amri M., Barry M. (2020). The psychological impact of COVID-19 pandemic on health care workers in a MERS-CoV endemic country. J. Infect. Public Health.

[18] Salazar de Pablo G., Vaquerizo-Serrano J., Catalan A., Arango C., Moreno C., Ferre F., Shin J.I., Sullivan S., Brondino N., Solmi M. (2020). Impact of coronavirus syndromes on physical and mental health of health care workers: Systematic review and meta-analysis. J. Affect. Disord..

[19] Li Y., Scherer N., Felix L., Kuper H. (2021). Prevalence of depression, anxiety and post-traumatic stress disorder in health care workers during the COVID-19 pandemic: A systematic review and meta-analysis. PLoS ONE.

[20] Batra K., Singh T.P., Sharma M., Batra R., Schvaneveldt N. (2020). Investigating the Psychological Impact of COVID-19 among Healthcare Workers: A Meta-Analysis. Int. J. Environ. Res. Public Health.

[21] Moher D., Liberati A., Tetzlaff J., Altman D.G. (2009). Preferred reporting items for systematic reviews and meta-analyses: The PRISMA statement. Ann. Intern. Med..

[22] Aromataris E., Munn Z. (2020). JBI Manual for Evidence Synthesis.

[23] Munn Z., Moola S., Lisy K., Riitano D., Tufanaru C. (2015). Methodological guidance for systematic reviews of observational epidemiological studies reporting prevalence and cumulative incidence data. Int. J. Evid. Based Healthc..

[24] Higgins J., Thomas J., Chandler J., Cumpston M., Li T., Page M., Welch V. (2021). Cochrane Handbook for Systematic Reviews of Interventions Version 6.2 (Updated February 2021).

[25] Borenstein M., Higgins J.P.T. (2013). Meta-Analysis and Subgroups. Prev. Sci..

[26] An Y., Sun Y., Liu Z., Chen Y. (2021). Investigation of the mental health status of frontier-line and non-frontier-line medical staff during a stress period. J. Affect. Disord..

[27] Awano N., Oyama N., Akiyama K., Inomata M., Kuse N., Tone M., Takada K., Muto Y., Fujimoto K., Akagi Y. (2020). Anxiety, Depression, and Resilience of Healthcare Workers in Japan During the Coronavirus Disease 2019 Outbreak. Intern. Med..

[28] Cai Z., Cui Q., Liu Z., Li J., Gong X., Liu J., Wan Z., Yuan X., Li X., Chen C. (2020). Nurses endured high risks of psychological problems under the epidemic of COVID-19 in a longitudinal study in Wuhan China. J. Psychiatr. Res..

[29] Chen J., Liu X., Wang D., Jin Y., He M., Ma Y., Zhao X., Song S., Zhang L., Xiang X. (2021). Risk factors for depression and anxiety in healthcare workers deployed during the COVID-19 outbreak in China. Soc. Psychiatry Psychiatr. Epidemiol..

[30] Guo W.-P., Min Q., Gu W.-W., Yu L., Xiao X., Yi W.-B., Li H.-L., Huang B., Li J.-L., Dai Y.-J. (2021). Prevalence of mental health problems in frontline healthcare workers after the first outbreak of COVID-19 in China: A cross-sectional study. Health Qual. Life Outcomes.

[31] Han L., Wong F.K.Y., She D.L.M., Li S.Y., Yang Y.F., Jiang M.Y., Ruan Y., Su Q., Ma Y., Chung L.Y.F. (2020). Anxiety and Depression of Nurses in a North West Province in China During the Period of Novel Coronavirus Pneumonia Outbreak. J. Nurs. Scholarsh..

[32] Hong S., Ai M., Xu X., Wang W., Chen J., Zhang Q., Wang L., Kuang L. (2021). Immediate psychological impact on nurses working at 42 government-designated hospitals during COVID-19 outbreak in China: A cross-sectional study. Nurs. Outlook.

[33] Huang L., Wang Y., Liu J., Ye P., Chen X., Xu H., Qu H., Ning G. (2020). Factors Influencing Anxiety of Health Care Workers in the Radiology Department with High Exposure Risk to COVID-19. Med. Sci. Monit. Int. Med. J. Exp. Clin. Res..

[34] Huang Y., Zhao N. (2021). Mental health burden for the public affected by the COVID-19 outbreak in China: Who will be the high-risk group?. Psychol. Health Med..

[35] Li J., Xu J., Zhou H., You H., Wang X., Li Y., Liang Y., Li S., Ma L., Zeng J. (2021). Working conditions and health status of 6,317 front line public health workers across five provinces in China during the COVID-19 epidemic: A cross-sectional study. BMC Public Health.

[36] Li R., Chen Y., Lv J., Liu L., Zong S., Li H., Li H. (2020). Anxiety and related factors in frontline clinical nurses fighting COVID-19 in Wuhan. Medicine.

[37] Li X., Li S., Xiang M., Fang Y., Qian K., Xu J., Li J., Zhang Z., Wang B. (2020). The prevalence and risk factors of PTSD symptoms among medical assistance workers during the COVID-19 pandemic. J. Psychosom. Res..

[38] Liang Y., Wu K., Zhou Y., Huang X., Zhou Y., Liu Z. (2020). Mental Health in Frontline Medical Workers during the 2019 Novel Coronavirus Disease Epidemic in China: A Comparison with the General Population. Int. J. Environ. Res. Public Health.

[39] Liu Y., Chen H., Zhang N., Wang X., Fan Q., Zhang Y., Huang L., Hu B., Li M. (2021). Anxiety and depression symptoms of medical staff under COVID-19 epidemic in China. J. Affect. Disord..

[40] Lu P., Li X., Lu L., Zhang Y. (2020). The psychological states of people after Wuhan eased the lockdown. PLoS ONE.

[41] Lu W., Wang H., Lin Y., Li L. (2020). Psychological status of medical workforce during the COVID-19 pandemic: A cross-sectional study. Psychiatry Res..

[42] Ning X., Yu F., Huang Q., Li X., Luo Y., Huang Q., Chen C. (2020). The mental health of neurological doctors and nurses in Hunan Province, China during the initial stages of the COVID-19 outbreak. BMC Psychiatry.

[43] Pan X., Xiao Y., Ren D., Xu Z.-M., Zhang Q., Yang L.-Y., Liu F., Hao Y.-S., Zhao F., Bai Y.-H. (2020). Prevalence of mental health problems and associated risk factors among military healthcare workers in specialized COVID-19 hospitals in Wuhan, China: A cross-sectional survey. Asia-Pac. Psychiatry.

[44] Pang Y., Fang H., Li L., Chen M., Chen Y., Chen M. (2021). Predictive factors of anxiety and depression among nurses fighting coronavirus disease 2019 in China. Int. J. Ment. Health Nurs..

[45] Park C., Hwang J.M., Jo S., Bae S.J., Sakong J. (2020). COVID-19 Outbreak and Its Association with Healthcare Workers’ Emotional Stress: A Cross-Sectional Study. J. Korean Med. Sci..

[46] Park S.Y., Kim B., Jung D.S., Jung S.I., Oh W.S., Kim S.W., Peck K.R., Chang H.H. (2020). Psychological distress among infectious disease physicians during the response to the COVID-19 outbreak in the Republic of Korea. BMC Public Health.

[47] Si M.Y., Su X.Y., Jiang Y., Wang W.J., Gu X.F., Ma L., Li J., Zhang S.K., Ren Z.F., Ren R. (2020). Psychological impact of COVID-19 on medical care workers in China. Infect. Dis. Poverty.

[48] Song X., Fu W., Liu X., Luo Z., Wang R., Zhou N., Yan S., Lv C. (2020). Mental health status of medical staff in emergency departments during the Coronavirus disease 2019 epidemic in China. Brain Behav. Immun..

[49] Tu Z.H., He J.W., Zhou N. (2020). Sleep quality and mood symptoms in conscripted frontline nurse in Wuhan, China during COVID-19 outbreak: A cross-sectional study. Medicine.

[50] Wang L.Q., Zhang M., Liu G.M., Nan S.Y., Li T., Xu L., Xue Y., Zhang M., Wang L., Qu Y.D. (2020). Psychological impact of coronavirus disease (2019) (COVID-19) epidemic on medical staff in different posts in China: A multicenter study. J. Psychiatr. Res..

[51] Wang M., Zhao Q., Hu C., Wang Y., Cao J., Huang S., Li J., Huang Y., Liang Q., Guo Z. (2021). Prevalence of psychological disorders in the COVID-19 epidemic in China: A real world cross-sectional study. J. Affect. Disord..

[52] Wang Y., Ma S., Yang C., Cai Z., Hu S., Zhang B., Tang S., Bai H., Guo X., Wu J. (2020). Acute psychological effects of Coronavirus Disease 2019 outbreak among healthcare workers in China: A cross-sectional study. Transl. Psychiatry.

[53] Xia Y., Zhang H., Xia Y., Li H., Zhai L., Wang H. (2021). The self-psychological safety maintenance and its influencing factors of community frontline staff during COVID-19 pandemic. Medicine.

[54] Xiao X., Zhu X., Fu S., Hu Y., Li X., Xiao J. (2020). Psychological impact of healthcare workers in China during COVID-19 pneumonia epidemic: A multi-center cross-sectional survey investigation. J. Affect. Disord..

[55] Xiaoming X., Ming A., Su H., Wo W., Jianmei C., Qi Z., Hua H., Xuemei L., Lixia W., Jun C. (2020). The psychological status of 8817 hospital workers during COVID-19 Epidemic: A cross-sectional study in Chongqing. J. Affect. Disord..

[56] Xing J., Sun N., Xu J., Geng S., Li Y. (2020). Study of the mental health status of medical personnel dealing with new coronavirus pneumonia. PLoS ONE.

[57] Xu X., Wang W., Chen J., Ai M., Shi L., Wang L., Hong S., Zhang Q., Hu H., Li X. (2021). Suicidal and self-harm ideation among Chinese hospital staff during the COVID-19 pandemic: Prevalence and correlates. Psychiatry Res..

[58] Zhan Y.X., Zhao S.Y., Yuan J., Liu H., Liu Y.F., Gui L.L., Zheng H., Zhou Y.M., Qiu L.H., Chen J.H. (2020). Prevalence and Influencing Factors on Fatigue of First-line Nurses Combating with COVID-19 in China: A Descriptive Cross-Sectional Study. Curr. Med. Sci..

[59] Zhang H., Shi Y., Jing P., Zhan P., Fang Y., Wang F. (2020). Posttraumatic stress disorder symptoms in healthcare workers after the peak of the COVID-19 outbreak: A survey of a large tertiary care hospital in Wuhan. Psychiatry Res..

[60] Zhao S., Cao J., Sun R., Zhang L., Liu B. (2020). Analysis of anxiety-related factors amongst frontline dental staff during the COVID-19 pandemic in Yichang, China. BMC Oral Health.

[61] Zheng R., Zhou Y., Fu Y., Xiang Q., Cheng F., Chen H., Xu H., Fu L., Wu X., Feng M. (2021). Prevalence and associated factors of depression and anxiety among nurses during the outbreak of COVID-19 in China: A cross-sectional study. Int. J. Nurs. Stud..

[62] Zhu W., Wei Y., Meng X., Li J. (2020). The mediation effects of coping style on the relationship between social support and anxiety in Chinese medical staff during COVID-19. BMC Health Serv. Res..

[63] Tan B.Y.Q., Kanneganti A., Lim L.J.H., Tan M., Chua Y.X., Tan L., Sia C.H., Denning M., Goh E.T., Purkayastha S. (2020). Burnout and Associated Factors Among Health Care Workers in Singapore During the COVID-19 Pandemic. J. Am. Med. Dir. Assoc..

[64] Sunjaya D.K., Herawati D.M.D., Siregar A.Y.M. (2021). Depressive, anxiety, and burnout symptoms on health care personnel at a month after COVID-19 outbreak in Indonesia. BMC Public Health.

[65] Mohd Fauzi M.F., Mohd Yusoff H., Muhamad Robat R., Mat Saruan N.A., Ismail K.I., Mohd Haris A.F. (2020). Doctors’ Mental Health in the Midst of COVID-19 Pandemic: The Roles of Work Demands and Recovery Experiences. Int. J. Environ. Res. Public Health.

[66] Tran T.V., Nguyen H.C., Pham L.V., Nguyen M.H., Nguyen H.C., Ha T.H., Phan D.T., Dao H.K., Nguyen P.B., Trinh M.V. (2020). Impacts and interactions of COVID-19 response involvement, health-related behaviours, health literacy on anxiety, depression and health-related quality of life among healthcare workers: A cross-sectional study. BMJ Open.

[67] Sim S.K., Lau B.L., Zaila S.R., Hazira N., Aniqah N.M., Panicker J., Hamzah A.S. (2021). Psychological symptoms among healthcare workers handling COVID-19 patients. Med. J. Malays..

[68] Amin F., Sharif S., Saeed R., Durrani N., Jilani D. (2020). COVID-19 pandemic- knowledge, perception, anxiety and depression among frontline doctors of Pakistan. BMC Psychiatry.

[69] Arshad A.R., Islam F. (2020). COVID-19 and Anxiety amongst Doctors: A Pakistani Perspective. J. Coll. Physicians Surg. Pak. JCPSP.

[70] Barua L., Zaman M.S., Omi F.R., Faruque M. (2020). Psychological burden of the COVID-19 pandemic and its associated factors among frontline doctors of Bangladesh: A cross-sectional study. F1000Research.

[71] Das A., Sil A., Jaiswal S., Rajeev R., Thole A., Jafferany M., Ali S.N. (2020). A Study to Evaluate Depression and Perceived Stress Among Frontline Indian Doctors Combating the COVID-19 Pandemic. Prim. Care Companion CNS Disord..

[72] Gupta B., Sharma V., Kumar N., Mahajan A. (2020). Anxiety and Sleep Disturbances Among Health Care Workers During the COVID-19 Pandemic in India: Cross-Sectional Online Survey. JMIR Public Health Surveill..

[73] Hassannia L., Taghizadeh F., Moosazadeh M., Zarghami M., Taghizadeh H., Dooki A.F., Fathi M., Alizadeh-Navaei R., Hedayatizadeh-Omran A., Dehghan N. (2021). Anxiety and Depression in Health Workers and General Population During COVID-19 in IRAN: A Cross-Sectional Study. Neuropsychopharmacol. Rep..

[74] Kafle K., Shrestha D.B., Baniya A., Lamichhane S., Shahi M., Gurung B., Tandan P., Ghimire A., Budhathoki P. (2021). Psychological distress among health service providers during COVID-19 pandemic in Nepal. PLoS ONE.

[75] Khanal P., Devkota N., Dahal M., Paudel K., Joshi D. (2020). Mental health impacts among health workers during COVID-19 in a low resource setting: A cross-sectional survey from Nepal. Glob. Health.

[76] Khanna R.C., Honavar S.G., Metla A.L., Bhattacharya A., Maulik P.K. (2020). Psychological impact of COVID-19 on ophthalmologists-in-training and practising ophthalmologists in India. Indian J. Ophthalmol..

[77] Khatun M.F., Parvin M.F., Rashid M.M., Alam M.S., Kamrunnahar M., Talukder A., Rahman Razu S., Ward P.R., Ali M. (2021). Mental Health of Physicians During COVID-19 Outbreak in Bangladesh: A Web-Based Cross-Sectional Survey. Front. Public Health.

[78] Kumar D., Saghir T., Ali G., Yasin U., Furnaz S., Karim M., Hussain M., Kumari R., Bai R., Kumar H. (2021). Psychosocial Impact of COVID-19 on Healthcare Workers at a Tertiary Care Cardiac Center of Karachi Pakistan. J. Occup. Environ. Med..

[79] Moayed M.S., Vahedian-Azimi A., Mirmomeni G., Rahimi-Bashar F., Goharimoghadam K., Pourhoseingholi M.A., Abbasi-Farajzadeh M., Hekmat M., Sathyapalan T., Guest P.C. (2021). Survey of Immediate Psychological Distress Levels Among Healthcare Workers in the COVID-19 Epidemic: A Cross-Sectional Study. Adv. Exp. Med. Biol..

[80] Mohammadian Khonsari N., Shafiee G., Zandifar A., Mohammad Poornami S., Ejtahed H.S., Asayesh H., Qorbani M. (2021). Comparison of psychological symptoms between infected and non-infected COVID-19 health care workers. BMC Psychiatry.

[81] Pandey A., Sharma C., Chapagain R.H., Devkota N., Ranabhat K., Pant S., Adhikari K. (2021). Stress, Anxiety, Depression and Their Associated Factors among Health Care Workers During COVID -19 Pandemic in Nepal. J. Nepal Health Res. Counc..

[82] Parthasarathy R., Ts J., K. T., Murthy P. (2021). Mental health issues among health care workers during the COVID-19 pandemic—A study from India. Asian J. Psychiatry.

[83] Perera B., Wickramarachchi B., Samanmalie C., Hettiarachchi M. (2021). Psychological experiences of healthcare professionals in Sri Lanka during COVID-19. BMC Psychol..

[84] Saeed R., Amin F., Talha M., Randenikumara S., Shariff I., Durrani N., Salman S. (2021). COVID-19 Pandemic Prevalence and Risk Factors for Depression among Health Care Workers in South Asia. Asia Pac. J. Public Health.

[85] Suryavanshi N., Kadam A., Dhumal G., Nimkar S., Mave V., Gupta A., Cox S.R., Gupte N. (2020). Mental health and quality of life among healthcare professionals during the COVID-19 pandemic in India. Brain Behav..

[86] AlAteeq D.A., Aljhani S., Althiyabi I., Majzoub S. (2020). Mental health among healthcare providers during coronavirus disease (COVID-19) outbreak in Saudi Arabia. J. Infect. Public Health.

[87] Alenazi T.H., BinDhim N.F., Alenazi M.H., Tamim H., Almagrabi R.S., Aljohani S.M., M H.B., Almubark R.A., Althumiri N.A., Alqahtani S.A. (2020). Prevalence and predictors of anxiety among healthcare workers in Saudi Arabia during the COVID-19 pandemic. J. Infect. Public Health.

[88] Almater A.I., Tobaigy M.F., Younis A.S., Alaqeel M.K., Abouammoh M.A. (2020). Effect of 2019 Coronavirus Pandemic on Ophthalmologists Practicing in Saudi Arabia: A Psychological Health Assessment. Middle East Afr. J. Ophthalmol..

[89] Alrubaiee G.G., Al-Qalah T.A.H., Al-Aawar M.S.A. (2020). Knowledge, attitudes, anxiety, and preventive behaviours towards COVID-19 among health care providers in Yemen: An online cross-sectional survey. BMC Public Health.

[90] Alsairafi Z., Naser A.Y., Alsaleh F.M., Awad A., Jalal Z. (2021). Mental Health Status of Healthcare Professionals and Students of Health Sciences Faculties in Kuwait during the COVID-19 Pandemic. Int. J. Environ. Res. Public Health.

[91] Alshekaili M., Hassan W., Al Said N., Al Sulaimani F., Jayapal S.K., Al-Mawali A., Chan M.F., Mahadevan S., Al-Adawi S. (2020). Factors associated with mental health outcomes across healthcare settings in Oman during COVID-19: Frontline versus non-frontline healthcare workers. BMJ Open.

[92] Arafa A., Mohammed Z., Mahmoud O., Elshazley M., Ewis A. (2021). Depressed, anxious, and stressed: What have healthcare workers on the frontlines in Egypt and Saudi Arabia experienced during the COVID-19 pandemic?. J. Affect. Disord..

[93] Balay-Odao E.M., Alquwez N., Inocian E.P., Alotaibi R.S. (2020). Hospital Preparedness, Resilience, and Psychological Burden Among Clinical Nurses in Addressing the COVID-19 Crisis in Riyadh, Saudi Arabia. Front. Public Health.

[94] Koksal E., Dost B., Terzi Ö., Ustun Y.B., Özdin S., Bilgin S. (2020). Evaluation of Depression and Anxiety Levels and Related Factors Among Operating Theater Workers During the Novel Coronavirus (COVID-19) Pandemic. J. Perianesthesia Nurs..

[95] Şahin M.K., Aker S., Şahin G., Karabekiroğlu A. (2020). Prevalence of Depression, Anxiety, Distress and Insomnia and Related Factors in Healthcare Workers During COVID-19 Pandemic in Turkey. J. Community Health.

[96] Yıldırım M., Özaslan A. (2021). Worry, Severity, Controllability, and Preventive Behaviours of COVID-19 and Their Associations with Mental Health of Turkish Healthcare Workers Working at a Pandemic Hospital. Int. J. Ment. Health Addict..

[97] Yilmaz A., Karakoyun D.O., Isik H.S., Bostan S. (2020). The Effect of the COVID-19 Pandemic on Functioning of Neurosurgery Clinics and the Anxiety Levels of Neurosurgeons in Turkey. Turk. Neurosurg..

[98] Yörük S., Güler D. (2021). The relationship between psychological resilience, burnout, stress, and sociodemographic factors with depression in nurses and midwives during the COVID-19 pandemic: A cross-sectional study in Turkey. Perspect. Psychiatr. Care.

[99] Chew N.W.S., Lee G.K.H., Tan B.Y.Q., Jing M., Goh Y., Ngiam N.J.H., Yeo L.L.L., Ahmad A., Ahmed Khan F., Napolean Shanmugam G. (2020). A multinational, multicentre study on the psychological outcomes and associated physical symptoms amongst healthcare workers during COVID-19 outbreak. Brain Behav. Immun..

[100] Chew N.W.S., Ngiam J.N., Tan B.Y.-Q., Tham S.-M., Tan C.Y.-S., Jing M., Sagayanathan R., Chen J.T., Wong L.Y.H., Ahmad A. (2020). Asian-Pacific perspective on the psychological well-being of healthcare workers during the evolution of the COVID-19 pandemic. BJPsych Open.

[101] Neto M.L.R., Almeida H.G., Esmeraldo J.D., Nobre C.B., Pinheiro W.R., de Oliveira C.R.T., Sousa I.D.C., Lima O., Lima N.N.R., Moreira M.M. (2020). When health professionals look death in the eye: The mental health of professionals who deal daily with the 2019 coronavirus outbreak. Psychiatry Res..

[102] Krishnamoorthy Y., Nagarajan R., Saya G.K., Menon V. (2020). Prevalence of psychological morbidities among general population, healthcare workers and COVID-19 patients amidst the COVID-19 pandemic: A systematic review and meta-analysis. Psychiatry Res..

[103] Al Maqbali M., Al Sinani M., Al-Lenjawi B. (2021). Prevalence of stress, depression, anxiety and sleep disturbance among nurses during the COVID-19 pandemic: A systematic review and meta-analysis. J. Psychosom. Res..

[104] Lai J., Ma S., Wang Y., Cai Z., Hu J., Wei N., Wu J., Du H., Chen T., Li R. (2020). Factors associated with mental health outcomes among health care workers exposed to Coronavirus Disease 2019. JAMA Netw. Open.

[105] Zhang C., Yang L., Liu S., Ma S., Wang Y., Cai Z., Du H., Li R., Kang L., Su M. (2020). Survey of Insomnia and Related Social Psychological Factors Among Medical Staff Involved in the 2019 Novel Coronavirus Disease Outbreak. Front Psychiatry.

[106] Okajima I., Chung S., Suh S. (2021). Validation of the Japanese-version Stress and Anxiety to Viral Epidemics-9 (SAVE-9) and relationship among stress, insomnia, anxiety, and depression in healthcare workers exposed to coronavirus disease 20191. Sleep Med..

[107] Chang M.C., Park D. (2020). Incidence of Post-Traumatic Stress Disorder after Coronavirus Disease. Healthcare.

[108] Pappa S., Chen J., Barnet J., Chang A., Dong R.K., Xu W., Yin A., Chen B.Z., Delios A., Chen R.Z. (2021). A Systematic Review and Meta-Analysis of the Mental Health Symptoms during the Covid-19 Pandemic in Southeast Asia. MedRxiv.

[109] Busch I.M., Moretti F., Mazzi M., Wu A.W., Rimondini M. (2021). What We Have Learned from Two Decades of Epidemics and Pandemics: A Systematic Review and Meta-Analysis of the Psychological Burden of Frontline Healthcare Workers. Psychother. Psychosom..

[110] Mohd Noor N., Che Yusof R., Yacob M.A. (2021). Anxiety in Frontline and Non-Frontline Healthcare Providers in Kelantan, Malaysia. Int. J. Environ. Res. Public Health.

[111] Tan B.Y.Q., Chew N.W.S., Lee G.K.H., Jing M., Goh Y., Yeo L.L.L., Zhang K., Chin H.K., Ahmad A., Khan F.A. (2020). Psychological impact of the COVID-19 pandemic on health care workers in Singapore. Ann. Intern. Med..

[112] Chou W.-P., Wang P.-W., Chen S.-L., Chang Y.-P., Wu C.-F., Lu W.-H., Yen C.-F. (2020). Risk Perception, Protective Behaviors, and General Anxiety during the Coronavirus Disease 2019 Pandemic among Affiliated Health Care Professionals in Taiwan: Comparisons with Frontline Health Care Professionals and the General Public. Int. J. Environ. Res. Public Health.

[113] Danet Danet A. (2021). Psychological impact of COVID-19 pandemic in Western frontline healthcare professionals. A systematic review. Med. Clínica.

[114] Shaukat N., Ali D.M., Razzak J. (2020). Physical and mental health impacts of COVID-19 on healthcare workers: A scoping review. Int. J. Emerg. Med..

[115] Labrague L.J., De Los Santos J.A.A. (2020). COVID-19 anxiety among front-line nurses: Predictive role of organisational support, personal resilience and social support. J. Nurs. Manag..

