# Supplementary material for: Prevalence of Psychological Impacts on Healthcare Providers during COVID-19 Pandemic in Asia
Source: Int J Environ Res Public Health. 2021 Aug 30;18(17):9157. doi: 10.3390/ijerph18179157 (PMC8431592; doi:10.3390/ijerph18179157)
Supplement: Supplementary file 1 [file ijerph-18-09157-s001.zip › Table S1.pdf]

Table SI: JBI Quality Assessment Tool for prevalence studies

| **Criteria (Yes/No/Unclear/ NA)** | Appropriate sample frame | Appropriate sampling participant | Adequate sample size | Description of subjects and the setting | Provide sample size justification, power description, or variance and effect estimates | Valid method | Condition measured in a standard, reliable way for all | Appropriate statistical analysis | Adequate response rate | Total quality score | Risk of Bias |
|---|---|---|---|---|---|---|---|---|---|---|---|
| Alateeq et al, 2020 | 1 | 1 | 1 | 1 | 1 | 1 | 1 | 1 | 1 | 9 | low |
| Alenazi et al, 2020 | 1 | 1 | 1 | 1 | 1 | 1 | 1 | 1 | 1 | 9 | low |
| Almater et al., 2020 | 1 | 1 | 0 | 1 | 0 | 1 | 1 | 1 | 0 | 6 | moderate |
| Alrubaiee et al., 2020 | 0 | 0 | 0 | 1 | 0 | 1 | 1 | 1 | 0 | 4 | high |
| Alsairafi et al., 2021 | 1 | 1 | 1 | 1 | 1 | 1 | 1 | 1 | 1 | 9 | low |
| Alshekaili et al., 2020 | 1 | 1 | 1 | 1 | 1 | 1 | 1 | 1 | 1 | 9 | low |
| Amin et al., 2020 | 1 | 0 | 1 | 1 | 1 | 1 | 1 | 1 | 1 | 8 | low |
| An et al., 2021 | 0 | 0 | 0 | 1 | 0 | 1 | 1 | 1 | 0 | 4 | high |
| Arafa et al., 2021 | 1 | 1 | 0 | 1 | 0 | 1 | 1 | 1 | 0 | 6 | moderate |
| Arshad & islam, 2020 | 1 | 1 | 0 | 1 | 0 | 1 | 1 | 1 | 0 | 6 | moderate |
| Awano et al., 2020 | 1 | 1 | 0 | 1 | 0 | 1 | 1 | 1 | 0 | 6 | moderate |
| Balay et al., 2020 | 1 | 1 | 1 | 1 | 1 | 1 | 1 | 1 | 1 | 9 | low |
| Barua et al., 2020 | 0 | 1 | 1 | 1 | 0 | 1 | 1 | 1 | 1 | 7 | low |
| Cai q et al., 2020 | 0 | 1 | 0 | 1 | 0 | 1 | 1 | 1 | 0 | 5 | moderate |
| Cai z et al., 2020 | 1 | 1 | 0 | 1 | 0 | 1 | 1 | 1 | 0 | 6 | moderate |
| Chen et al, 2021 | 0 | 1 | 0 | 0 | 0 | 1 | 1 | 1 | 0 | 4 | high |
| Chew et al., 2020 | 1 | 0 | 0 | 1 | 0 | 1 | 1 | 1 | 0 | 5 | moderate |
| Chew n et al., 2020 | 1 | 0 | 0 | 1 | 0 | 1 | 1 | 1 | 0 | 5 | moderate |
| Das et al, 2002 | 1 | 1 | 0 | 1 | 0 | 1 | 1 | 1 | 0 | 6 | moderate |
| Guo et al., 2021 | 1 | 1 | 0 | 1 | 0 | 1 | 1 | 1 | 0 | 6 | moderate |
| Gupta et al., 2020 | 1 | 1 | 0 | 1 | 0 | 1 | 1 | 1 | 0 | 6 | moderate |

| | | | | | | | | | | | |
|---|---|---|---|---|---|---|---|---|---|---|---|
| Han et al., 2020 | 1 | 1 | 0 | 1 | 0 | 1 | 1 | 1 | 0 | 6 | moderate |
| Hassannia et al, 2021 | 0 | 0 | 0 | 1 | 0 | 1 | 1 | 1 | 0 | 4 | high |
| Hong s et al., 2021 | 1 | 1 | 0 | 1 | 0 | 1 | 1 | 1 | 0 | 6 | moderate |
| Huang l et al., 2020 | 1 | 1 | 1 | 1 | 1 | 1 | 1 | 1 | 1 | 9 | low |
| Huang y et al., 2021 | 0 | 0 | 0 | 0 | 0 | 1 | 1 | 1 | 0 | 3 | high |
| Kafle et al., 2021 | 0 | 0 | 1 | 1 | 1 | 1 | 1 | 1 | 1 | 7 | low |
| Khanal et al., 2020 | 1 | 1 | 0 | 1 | 0 | 1 | 1 | 1 | 0 | 6 | moderate |
| Khanna et al., 2020 | 1 | 0 | 0 | 1 | 0 | 1 | 1 | 1 | 0 | 5 | moderate |
| Khatun et al., 2002 | 1 | 1 | 0 | 1 | 0 | 1 | 1 | 1 | 0 | 6 | moderate |
| Koksal et al., 2020 | 0 | 0 | 1 | 1 | 1 | 1 | 1 | 1 | 1 | 7 | low |
| Kumar et al., 2021 | 1 | 0 | 0 | 1 | 0 | 1 | 1 | 1 | 0 | 5 | moderate |
| Li j et al., 2021 | 1 | 1 | 1 | 1 | 0 | 1 | 1 | 1 | 1 | 8 | low |
| Li r et al., 2020 | 1 | 1 | 0 | 1 | 0 | 1 | 1 | 1 | 0 | 6 | moderate |
| Li x et al., 2020 | 1 | 0 | 0 | 1 | 0 | 1 | 1 | 1 | 0 | 5 | moderate |
| Liang et al., 2002 | 0 | 0 | 0 | 1 | 0 | 1 | 1 | 1 | 0 | 4 | high |
| Liu c et al., 2020 | 1 | 0 | 0 | 1 | 0 | 1 | 1 | 1 | 0 | 5 | moderate |
| Liu y et al., 2021 | 1 | 1 | 0 | 1 | 0 | 1 | 1 | 1 | 0 | 6 | moderate |
| Lu peixin et al., 2020 | 1 | 0 | 0 | 1 | 0 | 1 | 1 | 1 | 0 | 5 | moderate |
| Lu w et al., 2020 | 1 | 0 | 0 | 1 | 0 | 1 | 1 | 1 | 0 | 5 | moderate |
| Moayed et al., 2021 | 1 | 1 | 1 | 1 | 1 | 1 | 1 | 1 | 1 | 9 | low |
| Mohammadian khonsari et al., 2021 | 1 | 1 | 1 | 1 | 1 | 1 | 1 | 1 | 1 | 9 | low |
| Mohd fauzi et al., 2020 | 1 | 0 | 1 | 1 | 1 | 1 | 1 | 1 | 1 | 8 | low |
| Naser et al., 2020 | 0 | 1 | 1 | 1 | 1 | 1 | 1 | 1 | 1 | 8 | low |
| Ning x et al., 2020 | 1 | 1 | 0 | 1 | 0 | 1 | 1 | 1 | 0 | 6 | moderate |
| Pan et al., 2020 | 1 | 1 | 0 | 1 | 0 | 1 | 1 | 1 | 0 | 6 | moderate |
| Pandey et al., 2021 | 1 | 1 | 0 | 1 | 0 | 1 | 1 | 1 | 0 | 6 | moderate |
| Pang et al., 2021 | 1 | 1 | 0 | 1 | 0 | 1 | 1 | 1 | 0 | 6 | moderate |
| Park c et al., 2020 | 1 | 0 | 0 | 1 | 0 | 1 | 1 | 1 | 0 | 5 | moderate |
| Park s et al., 2020 | 1 | 0 | 0 | 1 | 0 | 1 | 1 | 1 | 0 | 5 | moderate |

| | | | | | | | | | | | |
|---|---|---|---|---|---|---|---|---|---|---|---|
| Parthasarathy et al., 2021 | 1 | 0 | 0 | 1 | 0 | 1 | 1 | 1 | 0 | 5 | moderate |
| Perera et al., 2021 | 1 | 0 | 0 | 1 | 0 | 1 | 1 | 1 | 0 | 5 | moderate |
| Saeed et al., 2021 | 0 | 0 | 0 | 1 | 0 | 1 | 1 | 1 | 0 | 4 | high |
| Sahin et al., 2020 | 0 | 0 | 1 | 0 | 1 | 1 | 1 | 1 | 1 | 6 | moderate |
| Si et al., 2020 | 1 | 0 | 0 | 1 | 0 | 1 | 1 | 1 | 0 | 5 | moderate |
| Sim et al., 2021 | 1 | 1 | 1 | 1 | 1 | 1 | 1 | 1 | 1 | 9 | low |
| Song et al., 2020 | 1 | 1 | 0 | 1 | 0 | 1 | 1 | 1 | 0 | 6 | moderate |
| Sunjaya et al., 2021 | 0 | 1 | 0 | 1 | 0 | 1 | 1 | 1 | 0 | 5 | moderate |
| Suryavanshi et al., 2020 | 1 | 1 | 0 | 1 | 0 | 1 | 1 | 1 | 0 | 6 | moderate |
| Tan et al., 2020 | 1 | 0 | 0 | 1 | 0 | 1 | 1 | 1 | 0 | 5 | moderate |
| Temsah et al., 2020 | 1 | 0 | 0 | 1 | 0 | 1 | 1 | 1 | 0 | 5 | moderate |
| Trans et al., 2020 | 1 | 1 | 0 | 1 | 0 | 1 | 1 | 1 | 0 | 6 | moderate |
| Tu et al., 2020 | 1 | 1 | 0 | 1 | 0 | 1 | 1 | 1 | 0 | 6 | moderate |
| Wang l et al., 2020 | 1 | 0 | 0 | 1 | 0 | 1 | 1 | 1 | 0 | 5 | moderate |
| Wang m et al., 2021 | 0 | 0 | 0 | 1 | 0 | 1 | 1 | 1 | 0 | 4 | high |
| Wang y et al., 2020 | 1 | 0 | 1 | 1 | 1 | 1 | 1 | 1 | 1 | 8 | low |
| Xia et al., 2021 | 0 | 0 | 0 | 1 | 0 | 1 | 1 | 1 | 0 | 4 | high |
| Xiao et al., 2020 | 1 | 0 | 0 | 0 | 0 | 1 | 1 | 1 | 0 | 4 | high |
| Xiaoming, 2020 | 1 | 1 | 0 | 1 | 0 | 1 | 1 | 1 | 0 | 6 | moderate |
| Xing et al., 2020 | 1 | 1 | 0 | 1 | 0 | 1 | 1 | 1 | 0 | 6 | moderate |
| Xu et al., 2021 | 1 | 1 | 0 | 1 | 0 | 1 | 1 | 1 | 0 | 6 | moderate |
| Yang s et al., 2020 | 1 | 1 | 0 | 1 | 0 | 1 | 1 | 1 | 0 | 6 | moderate |
| Yildrim et al., 2021 | 1 | 1 | 0 | 1 | 0 | 1 | 1 | 1 | 0 | 6 | moderate |
| Yilmaz et al., 2020 | 1 | 0 | 0 | 1 | 0 | 1 | 1 | 1 | 0 | 5 | moderate |
| Yoruk & guler, 2021 | 0 | 0 | 1 | 0 | 1 | 1 | 1 | 1 | 1 | 6 | moderate |
| Zhan et al., 2020 | 1 | 1 | 1 | 1 | 1 | 1 | 1 | 1 | 1 | 9 | low |
| Zhang et al., 2020 | 1 | 0 | 0 | 1 | 0 | 1 | 1 | 1 | 0 | 5 | moderate |
| Zhao et al., 2020 | 0 | 0 | 0 | 1 | 0 | 1 | 1 | 1 | 0 | 4 | high |

| | | | | | | | | | | | |
|---|---|---|---|---|---|---|---|---|---|---|---|
| Zheng et al., 2021 | 1 | 1 | 0 | 1 | 0 | 1 | 1 | 1 | 0 | 6 | moderate |
| Zhu et al., 2020 | 1 | 1 | 0 | 1 | 0 | 1 | 1 | 1 | 0 | 6 | moderate |
