# Supplementary material for: Prevalence of Psychological Impacts on Healthcare Providers during COVID-19 Pandemic in Asia
Source: Int J Environ Res Public Health. 2021 Aug 30;18(17):9157. doi: 10.3390/ijerph18179157 (PMC8431592; doi:10.3390/ijerph18179157)
Supplement: Supplementary file 1 [file ijerph-18-09157-s001.zip › Table S2.pdf]

Table S2: Studies characteristics

Author	Year	Country	Region	Tool
AlAteeq et. al.	2020	Saudi Arabia	Western Asia	PHQ-9, GAD-7
Alenazi et. al.	2020	Saudi Arabia	Western Asia	The one-item question Likert scale for anxiety
Almater et. al.	2020	Saudi Arabia	Western Asia	PHQ-9, GAD-7, ISI, PSS-10
Alrubaiee et. al.	2020	Yemen	Western Asia	The respondents' anxiety (17-items)
Alsairafi et. al.	2021	Kuwait	Western Asia	PHQ-9, GAD-7
Alshekaili et. al.	2020	Oman	Western Asia	DASS-21, ISI
Amin et. al.	2020	Pakistan	Southern Asia	SRQ-20
An et. al.	2021	China	Eastern Asia	The Chinese version of CPSS, 20-item SDS. 20-item SAS,
Arafa et. al.	2021	Saudi Arabia	Western Asia	DASS-21
Arshad & Islam	2020	Pakistan	Southern Asia	GAD-7
Awano et. al.	2020	Japan	Eastern Asia	The Japanese versions of the GAD-7, CES-D
Balay et. al.	2020	Saudi Arabia	Western Asia	DASS-21
Barua et. al.	2020	Bangladesh	Southern Asia	PHQ-4
Cai Q et. al.	2020	China	Eastern Asia	BAI, ISI, PHQ-9
Cai Z et. al.	2020	China	Eastern Asia	PHQ-9, GAD-7, ISI, IESR
Chen et. al.	2021	China	Eastern Asia	GAD-7, PHQ-9
Chew et. al.	2020	India, Indonesia, Singapore, Malaysia, Vietnam	Southern Asia and South-Eastern Asia	DASS-21, IESR
Chew N et. al.	2020	Singapore, India	Southern Asia and South-Eastern Asia	DASS-21, IESR
Das et. al.	2020	India	Southern Asia	PHQ-9, PSS-10
Guo et. al.	2021	China	Eastern Asia	GAD-7, PSQI, PHQ-9, PCL-C
Gupta et. al.	2020	India	Southern Asia	GAD-7, single-item sleep quality scale
Han et. al.	2020	China	Eastern Asia	SAS, SDS
Hassannia et. al.	2021	Iran	Southern Asia	HADS
Hong et. al.	2021	China	Eastern Asia	A job-related stress severity scale developed for frontline health care workers in severe acute respiratory syndrome

				(SARS) outbreak, PHQ-9, GAD-7
Huang L et. al.	2020	China	Eastern Asia	SAS
Huang Y et. al.	2021	China	Eastern Asia	Chinese version of GAD-7, CES-D, PSQI
Kafle et. al.	2021	Nepal	Southern Asia	CPDI
Khanal et. al.	2020	Nepal	Southern Asia	HADS, ISI
Khanna et. al.	2020	India	Southern Asia	PHQ-9
Khatun et. al.	2021	Bangladesh	Southern Asia	GAD-7, PHQ-9
Koksal et. al.	2020	Turkey	Western Asia	HADS
Kumar et. al.	2021	Pakistan	Southern Asia	DASS-21
Li J et. al.	2021	China	Eastern Asia	PHQ-9, GAD
Li R et. al.	2020	China	Eastern Asia	HAMA
Li X et. al.	2020	China	Eastern Asia	IES-R, DASS-21
Liang et. al.	2020	China	Eastern Asia	PHQ-9, GAD-7, ISI
Liu C et. al.	2020	China	Eastern Asia	ZAS
Liu Y et. al.	2021	China	Eastern Asia	PSS-10, GAD-7, PHQ-9
Lu Peixin et. al.	2020	China	Eastern Asia	PHQ-9, GAD-7, PCL-C
Lu W et. al.	2020	China	Eastern Asia	HAMA, HAMD
Moayed et. al.	2021	Iran	Southern Asia	DASS-21
Mohammadian Khonsari et. al.	2021	Iran	Southern Asia	PTSD-8, DASS-21
Mohd Fauzi et. al.	2020	Malaysia	South-Eastern Asia	DASS-21
Naser et. al.	2020	Jordan	Western Asia	PHQ-9, GAD-7
Ning X et. al.	2020	China	Eastern Asia	The Chinese version of SAS, SDS
Pan et. al.	2020	China	Eastern Asia	PHQ-9, GAD-7, PHQ-15
Pandey et. al.	2021	Nepal	Southern Asia	DASS-21
Pang et. al.	2021	China	Eastern Asia	GAD-7, PHQ-9
Park C et. al.	2020	Korea	Eastern Asia	Korean version GAD
Park S et. al.	2020	Korea	Eastern Asia	DASS-21
Parthasarathy et. al.	2021	India	Southern Asia	PHQ-4
Perera et. al.	2021	Sri Lanka	Southern Asia	GAD-7, CES-D-revised-10
Saeed et. al.	2021	South Asia	Southern Asia	SRQ-20
Sahin et. al.	2020	Turkey	Western Asia	PHQ-9, GAD-7, ISI, IESR
Si et. al.	2020	China	Eastern Asia	IES-6, DASS-21, PCL-C
Sim et. al.	2021	Malaysia	South-Eastern Asia	DASS-21.
Song et. al.	2020	China	Eastern Asia	PSSS, CES-D), PCL-5
Sunjaya et. al.	2021	Indonesia	South-Eastern Asia	CESD R-10, ZAS
Suryavanshi et. al.	2020	India	Southern Asia	PHQ-9, GAD-7
Tan et. al.	2020	Singapore	South-Eastern Asia	HADS
Temsah et. al.	2020	Saudi Arabia	Western Asia	GAD-7

Tran et. al.	2020	Vietnam	South-Eastern Asia	GAD-7, PHQ-9
Tu et. al.	2020	China	Eastern Asia	PSQI, GAD-7, PHQ-9
Wang L et. al.	2020	China	Eastern Asia	GAD-7, PHQ-9, PSQI, PSS-14
Wang M et. al.	2021	China	Eastern Asia	PHQ-9, ISI, GAD-7
Wang Y et. al.	2020	China	Eastern Asia	PHQ-9, GAD-7, IESR
Xia et. al.	2021	China	Eastern Asia	ASI-3, PHQ-9
Xiao et. al.	2020	China	Eastern Asia	PSS-14, HADS.
Xiaoming	2020	China	Eastern Asia	PHQ-9, GAD-7, PHQ-15, and the score of stress and support scales.
Xing et. al.	2020	China	Eastern Asia	The symptom checklist-90
Xu et. al.	2021	China	Eastern Asia	PHQ-9, reformulated a 14-item perceived stress scale and a 6-item perceived support scale
Yang S et. al.	2020	Korea	Eastern Asia	GAD-7, PHQ-9
Yildrim et. al.	2021	Turkey	Western Asia	DASS-21
Yilmaz et. al.	2020	Turkey	Western Asia	BAI
Yoruk & Guler	2021	Turkey	Western Asia	PSS, BAI
Zhan et. al.	2020	China	Eastern Asia	GAD-7, PHQ-9, Chinese version PSS
Zhang et. al.	2020	China	Eastern Asia	PCL-C, HADS, PHQ-15, ISI
Zhao et. al.	2020	China	Eastern Asia	The Chinese version of BAI
Zheng et. al.	2021	China	Eastern Asia	SDS, SAS
Zhu et. al.	2020	China	Eastern Asia	SAS

ASI-3= Anxiety sensitivity index-3; BAI= The Beck anxiety inventory; CES-D= The center for epidemiologic studies depression scale; CESD R-10= The centre for epidemiological studies depression scale; CPDI= COVID-19 peritraumatic distress index; DASS-21= The depression, anxiety and stress scale; GAD-7= Seven-item generalized anxiety disorder 7; HADS= Hospital anxiety and depression scale; HAMA= The Hamilton rating scale for anxiety; HAMD= The Hamilton rating scale for depression; IES-6= Impact of events scale-6; IESR= Impact of events scale-revised; ISI= Insomnia severity index; PCL-5= PTSD checklist for dsm-5; PCL-C= PTSD checklist-civilian version; PHQ-4= Four-item Patient health questionnaire; PHQ-9= Nine-item Patient health questionnaire; PHQ-15= 15-item Patient health questionnaire; PSQI= Pittsburgh sleep quality index; PSS-10= 10-item perceived stress scale; PSS-14= 14-Perceived stress scale; PSSS= The perceived social support scale; PTSD-8= Post-traumatic stress disorder-8; SAS= Self-rating anxiety scale; SDS= Self-rating depression scale; SRQ-20= World health organization self-reporting questionnaire; ZAS= The Zung anxiety scale
